# Genetics and Genomics of Melanoma: Current Progress and Future Directions

**DOI:** 10.3390/genes14010232

**Published:** 2023-01-16

**Authors:** Camelia Quek

**Affiliations:** 1 Melanoma Institute Australia, The University of Sydney, Sydney, NSW 2006, Australia; camelia.quek@sydney.edu.au; 2 Faculty of Medicine and Health, The University of Sydney, Sydney, NSW 2006, Australia; 3 Charles Perkins Centre, The University of Sydney, Sydney, NSW 2006, Australia

## Introduction

Melanoma is a form of skin cancer that develops in the skin’s pigment cells, known as melanocytes, and can spread via blood and the lymphatic system to nearby tissues or distant organs in the body. Ultraviolet irradiation and mutations in the mitogen-activated protein kinase pathway and phosphatidylinositol 3-kinase/protein kinase B pathways have been identified as major driving forces in melanoma genesis. In the past few decades, the survival rates for patients with metastatic disease or high-risk early-stage disease have been historically poor. Recent immunotherapies such as immune checkpoint inhibitors have revolutionised cancer treatment and now being routinely used in a subgroup of cancer patients. This Special Issue, entitled “Genetics and Genomics of Melanoma”, focuses on the impact of genetics and genomics on our understanding and treatments of melanoma. This issue contains seven original research articles and four reviews that provide further insights to advance our knowledge of melanoma, as well as the development of diagnostic and therapeutic strategies to manage and treat melanoma.

Melanoma is a multi-factorial disease involving the accumulation of genetic abnormalities and is influenced by environmental and host factors. As reviewed by Motwani and Eccles [1], the molecular complexity of melanoma is not only driven by genomic alterations and transcriptomic dysregulation, but also by the impact of clonal evolution and cancer stem cell origin theory; these drive tumour heterogeneity and disease, leading to the progression of primary melanoma to invasion and metastasis. The authors further described the mutational landscape of melanoma such as CDK4, MITF, PTEN and BRAF. These mutations contribute to the phenotypic differences observed between invasive and non-invasive melanocytes. They also discussed the common oncogenic signalling pathways underlying melanoma initiation, adaptation and metastasis. This work provides a comprehensive review of genomic alterations and pathways associated with the invasion and metastasis of melanoma.

Of all solid tumours, melanoma has among the highest rate of mutated malignancies, harbouring both somatic and germline mutations. Here, we present three original research articles which focus on the impact of genetic mutations that can be strategically used for patient management and treatment decisions. Firstly, Tarazon et al. identified that miR-138-5p is a crucial tumour-suppressor microRNA involved in the regulation of hTERT protein [2]. The authors performed molecular and biochemistry assays to discover that miR-138-5p is capable of blocking hTERT translation and supressing cell growth, reflecting the respective decreased protein expression of hTERT protein and low cell viability. Next, Djulbegovi et al. reported that intrinsically disordered protein regions (IDPRs) and their protein–protein interactions (PPI) in subjects with mutations in BRAF, NRAS, c-KIT, NF1 or PTEN are associated with rare neoplasms including conjunctival melanoma [3]. Djulbegovi and colleagues performed bioinformatic predictions and interaction-network and pathway analyses to show that poor regulation of IDPRs along the key proteins (BRAF, NRAS, c-KIT, NF1 or PTEN) is likely to promote oncogenesis and neoplastic development. The overall work can be adapted to be used in functional genomic and proteomic research in order to develop novel drug targets or small-molecule inhibitors to assist with the management of patients with conjunctival melanoma. Thirdly, Vergani and colleagues addressed the occurrence of MITF-E318K variant and its association with germline CDKN2A and MC1R mutations on a clinical cohort comprising of 248 melanoma patients [4]. They found that the inherited genetic variation in MC1R genes was associated with an increased risk of melanoma, and CDKN2A pathogenic mutations were very rare or absent in this cohort. The authors also conducted an *in silico* analysis using TCGA Pan-Cancer-Atlas data to confirm that patients with MITF-E318K melanoma carriers harboured a common pattern of 9p21.3 deletion. The overall results provided greater insights into the clinical strategies that could be used to manage and treat MITF-associated tumour types.

Genetic mutations such as BAP1 are found in several subtypes of melanoma, and these include cutaneous melanocytic tumours and uveal melanoma. Two original research publications focused on the implications of BAP1 mutation in melanoma patients. Zhou et al. identified that the three lesions in a patient experiencing the loss of BAP1 expression played a role in upregulating the protein kinase pathway (ROS1 and NTRK3; [5]). The gene expression profiles revealed that MAPK-pathway-associated and gene-transcription factors including SOX10 and ETS1 were involved in tumour progression. The prognoses for patients with uveal melanoma with BAP1 mutation have been poor. Djulbegovic et al. demonstrated that the presence of intrinsically disordered protein regions (IDPRs) on the BAP1 protein are associated with uveal melanoma [6]. The authors found that the disordering patterns and ordered regions of the BAP1 protein may play a role in the development of uveal melanoma. Targeting IDPRs in BAP1 with small molecules will likely be considered as a novel drug development to manage patients with uveal melanoma.

Recent advances in next-generation technologies, automated robotics, computing and bioengineering in the industry have facilitated personalised or precision cancer medicine. Quek et al. and Bai et al. have written review papers that discuss how single-cell sequencing and spatial multi-omics help to further our understanding of checkpoint-based immunotherapies in terms of tumour-immune heterogeneity and their underlying mechanisms of response and resistance to treatment [7,8]. Quek and colleagues provided an example of the strategic use of specific single-cell platforms to characterise the tumour microenvironment and to identify immunotherapeutic-associated resistance mechanisms and cellular heterogeneity [8]. They also provided guidance for the implementation of single-cell analytics in clinical settings and discussed the opportunities surrounding the use of single-cell analyses to rationalise the design of novel drug developments. Furthermore, Bai and colleagues highlighted the potential of using single-cell spatial and sequencing approaches to dissect tumour heterogeneity and microenvironment complexities that drive drug resistance [7]. The authors discussed several potential resistant pathways including therapy-resistant BRAF cells, a loss of tumour-suppressor PTEN, impaired HLA-A regulation, AXL-high therapy-resistant cells and the MITF/AXL ratio. In addition to the clinical and industry applications of single-cell approaches in immune oncology, Olbryt and colleagues have demonstrated the potential of liquid biopsy genetic profiling to identify low- and high-frequency variants in melanoma samples using HD technology and the Ion Torrent platform [9]. The authors also highlighted the successful usage of Ion Torrent HD Technology to longitudinally monitor variants using cell-free DNA. Together, these studies reveal new insights into the applications of next-generation technologies in enabling biomarker and drug discovery with higher precision.

With an increased number of data sets generated from healthcare and genomic technologies, bioinformatics and machine learning can help with understanding the complexity of tumour microenvironment, disease heterogeneity, tumourigenesis and novel drug discovery for the prognosis, prediction and treatments of cancer. Ma et al. summarised the key genetic drivers (such as BRAF, PTEN, TERT, NRAS and CDKN2A) of melanoma and have provided applications of bioinformatics and machine learning models in the management of melanoma patients [10]. The authors stated that the gene expression profiles, clinicopathologic predictions (e.g., Breslow thickness and patient age) and genetic (somatic and germline) variants associated with melanoma metastasis, survival and mortality are important in melanoma risk-stratification tools. A robustly validated risk-stratification tool using bioinformatic and machine-learning models will facilitate clinical utilities in predicting a melanoma prognosis. As gene expression profiling using coding and non-coding RNAs has been increasingly popular in understanding tumour progression and treatments, Barbagallo et al. established a VECTOR database to store several features of RNAs; the database offers a comprehensive picture of competitive endogenous RNA networks in melanoma [11]. Barbagallo and colleagues adapted the Laravel model–view–controller framework to retrieve information regarding: (i) the expression correlation values of miRNA-mRNA, miRNA-lncRNA and lncRNA-mRNA pairs combined with predicted or validated RNA–RNA interactions from TCGA; (ii) the sequencing data of overlapping-sense and anti-sense strains; (iii) the correlation values of Transcription Factor (TF)-miRNA, TF-lncRNA and TF-mRNA pairs associated with ChiPseq data; and (iv) the expression data of miRNAs, lncRNAs and mRNAs in both uveal melanoma and physiological tissues [11]. VECTOR has enabled researchers worldwide to study RNA and the signalling networks in melanoma.

In summary, the collection of publications in this current Special Issue “Genetics and Genomics of Melanoma” covers a range of topics and provides greater insights to direct future genetics and genomics research in melanoma. We anticipate that this Special Issue will facilitate researchers to advance cancer research in terms of the novel development of biomarkers and drug targets to better treat and manage patients, with the objective of achieving zero deaths from melanoma.
